# Evaluation of a New Tubular Finger Oxygen-Enriched Oil Inside-Coated Dressing Device in Pediatric Patients Undergoing Distal Hypospadias Repair: A Prospective Randomized Clinical Trial Part II

**DOI:** 10.3389/fped.2021.638406

**Published:** 2021-03-02

**Authors:** Ciro Esposito, Vincenzo Coppola, Fulvia Del Conte, Mariapina Cerulo, Giovanni Esposito, Felice Crocetto, Marco Castagnetti, Antonio Calignano, Maria Escolino

**Affiliations:** ^1^Division of Pediatric Surgery, Federico II University of Naples, Naples, Italy; ^2^Department of Pharmacy, Federico II University of Naples, Naples, Italy; ^3^Division of Pediatric Urology, Medical University of Padua, Padua, Italy

**Keywords:** hypospadias, dressing, oxygen-enriched oily gel device, wound, complications, children

## Abstract

**Background:** This study was the second part of a prospective randomized clinical trial and aimed to evaluate the use of a tubular finger oxygen-enriched oil inside-coated dressing device and its effect on the post-operative outcome of children undergoing distal hypospadias repair.

**Methods:** A prospective single-blinded randomized clinical trial was carried out between September 2019 and September 2020. We included all patients with distal hypospadias, who received Snodgrass urethroplasty and preputioplasty. The patients were randomized in two groups according to the type of dressing: tubular finger oxygen-enriched oil inside-coated device (G1) and elastic net bandage with application of oxygen-enriched oil-based gel (G2). The patients were evaluated at 7, 14, 21, 30, and 60 post-operative day (POD).

**Results:** Sixty-four patients (median age 14 months) were included in the study and randomized in two groups, each of 32 patients. Post-operative preputial edema rate was significantly lower in G1 (3/32, 9.3%) compared with G2 (10/32, 31.2%) (*p* = 0.001). The median duration of preputial edema was significantly shorter in G1 compared with G2 (6 vs. 10.5 days) (*p* = 0.001). Penile diameter measurements at 4th, 7th, 14th POD proved that entity and duration of post-operative swelling were objectively decreased using the new dressing. The wound healing was significantly faster in G1 compared with G2 (14.2 vs. 18.5 days) (*p* = 0.001). The post-operative complications rate was significantly lower in G1 (0%) compared with G2 (3/32, 9.3%) (*p* = 0.001). Foreskin dehiscence occurred in two G2 patients (6.2%) whereas, breakdown of urethroplasty and preputioplasty occurred in one G2 patient (3.1%) due to scratching injuries. The dressing management was subjectively assessed by nurses to be easier in G1 patients compared with G2 ones (median score 1.2 vs. 3.5) (*p* = 0.001). The median treatment costs were significantly lower in G1 compared with G2 (55 vs. 87 eur) (*p* = 0.001). No adverse skin reactions occurred.

**Conclusions:** Post-operative dressing using tubular finger oxygen-enriched oil inside-coated device was highly effective, easy to manage, cheaper and associated with a lower rate of foreskin and urethral complications compared with the standard dressing method in pediatric patients undergoing distal hypospadias repair. It was also clinically safe without allergy or intolerance to the product.

## Introduction

Probably one of the most controversial aspects of hypospadias surgery is the post-operative dressing (1). Analyzing the international literature, no consensus emerged about this argument (2) and an enormous number of surgical techniques as well as type of dressings have been described for operative and post-operative management of pediatric patients affected by hypospadias (3–10).

The ideal dressing should be soft and mobile, adaptable to the penis during the child's movements but at the same time rigid to stabilize the penis during the dynamic changes occurring in physiological erections (11). It should also keep the penis clean, preventing the fecal and urinary contamination, that occurs constantly in infants under 2–3 years of age, who wear the diaper. In addition, the dressing should protect the penis from external injuries, secondary to child's scratching, occurring very frequently in the post-operative period. Regarding the dressing type, it is not only important the structure of the dressing but also its substrate (3–7). In children wearing the diaper, the penis is always wet. For this reason, it is important to cover the inner part of the dressing, that is in direct contact with the wound, with a product that improves healing and avoids infections. Finally, the dressing should be easy to manage by nurses and its change or removal should be comfortable for the child (12–14).

We recently published a prospective randomized clinical trial reporting the efficacy of oxygen-enriched oil-based gel associated with an elastic band dressing on the post-operative wound healing outcome of children undergoing distal hypospadias repair (15).

More recently, a special medical device in the form of a single-use tubular finger or toe stall, coated on the inside with oxygen-enriched oily gel, has been marketed for treatment of specific finger or toe wounds, including surgical wounds, superficial burns, traumatic wounds, ulcers. The shape of this device was specifically designed to fit the anatomical form of the fingers or toes. We decided to apply this device to surgical wound following hypospadias surgery, since its shape seemed to perfectly fit the anatomical form of the penis.

This article represented the second part of the cited prospective randomized clinical trial and aimed to evaluate the use of a new tubular finger oxygen-enriched oil inside-coated dressing device and its effect on the post-operative outcome of children undergoing distal hypospadias repair.

## Materials and Methods

The second part of this prospective single-blinded randomized clinical trial was carried out between September 2019 and September 2020 and included all patients with distal hypospadias, who underwent Snodgrass urethroplasty and preputioplasty. This study received the appropriate approval by the Institute Review Board (IRB) and the Ethics Committee. Written informed consent was obtained from all patients before surgery.

### Patients' Selection

The study included all patients aged <3 years with distal hypospadias, who received Snodgrass urethroplasty and preputioplasty in our surgical unit. Patients with proximal hypospadias or with distal hypospadias aged >3 years or receiving circumcision or toilet-trained at time of surgery were excluded from the study.

### Sample Size and Sampling Method

It was calculated that the minimum sample size to obtain, at 80% power and at type 1 error of 5%, an absolute difference in rates of at least 14%, should be 24 in each treatment arm, with a total number of 48. Adding an expected attrition rate of 30% (to account for eventual loss to follow-up), the calculated sample size came to 64, randomized to 32 participants in each arm.

Randomization and patient allocation were performed using simple random sampling method, which entailed an equal number of ballot papers pre-labeled with either tubular finger oxygen-enriched oil inside-coated device (G1) and elastic net bandage with application of oxygen-enriched oil-based gel (G2), sealed in similarly opaque envelopes and picked before the surgical procedure.

### Operative Technique

All the patients received Snodgrass urethroplasty and preputioplasty. All the surgical procedures were performed by two experienced surgeons. The same sutures and post-operative urinary diversion were adopted in all patients, as already described in the first part of the study. All the procedures were performed under general anesthesia. After degloving the penis and removing the chordees, the urethral plate was incised and tubularized over an 8 Fr Foley catheter. The reconstruction of the urethra was performed using a running suture of 6/0 monofilament polyglyconate suture followed by a second layer of interrupted stitches using the same suture. The urethra was then covered with a well vascularized subcutaneous dartos flap, that was fixed to the new urethra using interrupted mattress stitches. Glanuloplasty was then performed using 5/0 polyglyconate interrupted sutures. Preputioplasty was performed with a three-layer closure. All the patients had an 8 Fr Foley silicon catheter inside the urethra, that was managed using a double diaper layer.

### Post-operative Wound Management

**Figure 1:**
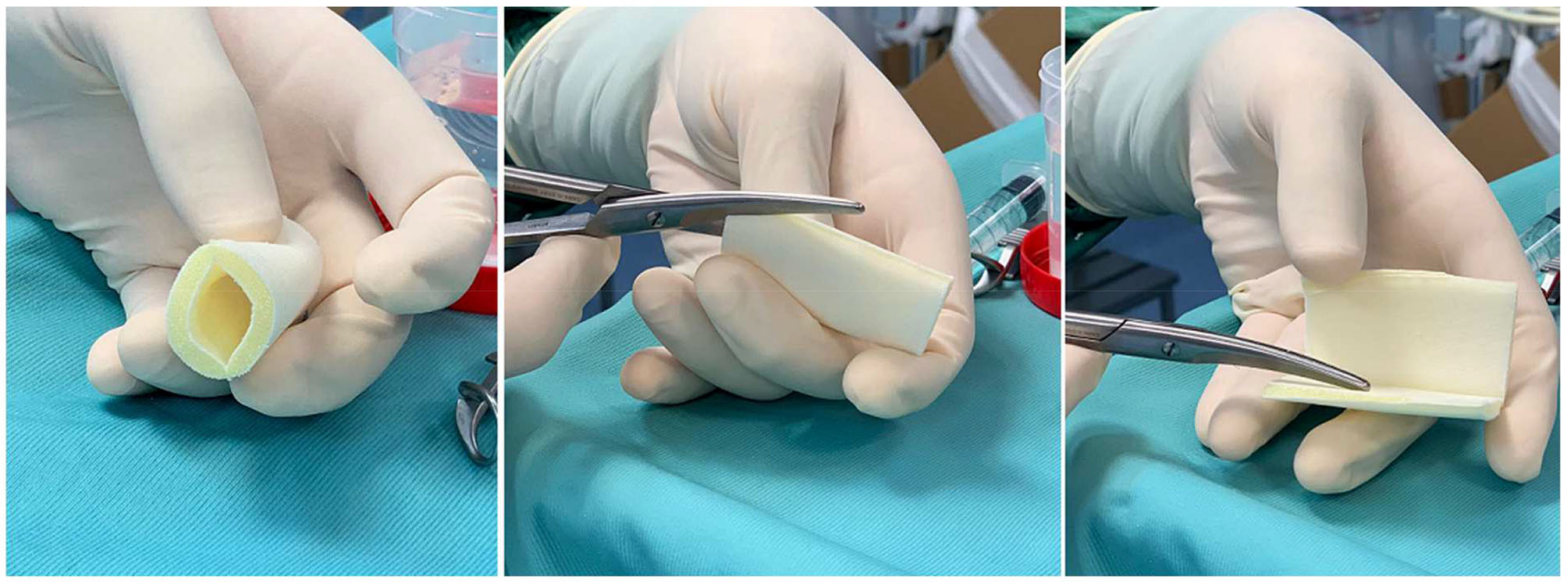
The device is reduced in length according to the penis' size and opened longitudinally.

**Figure 2:**
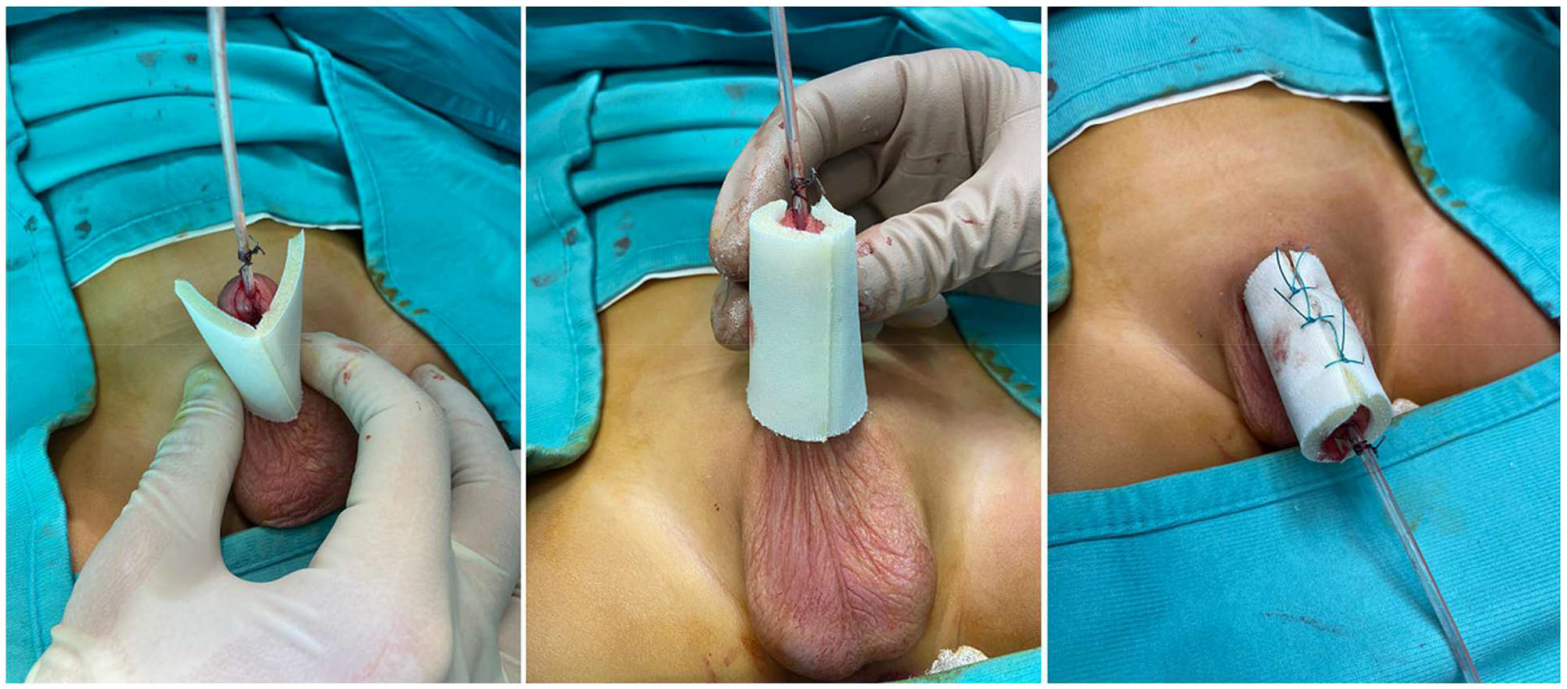
The device is applied onto the penis and stabilized on the dorsal aspect using 3 stitches.

**Figure 3:**
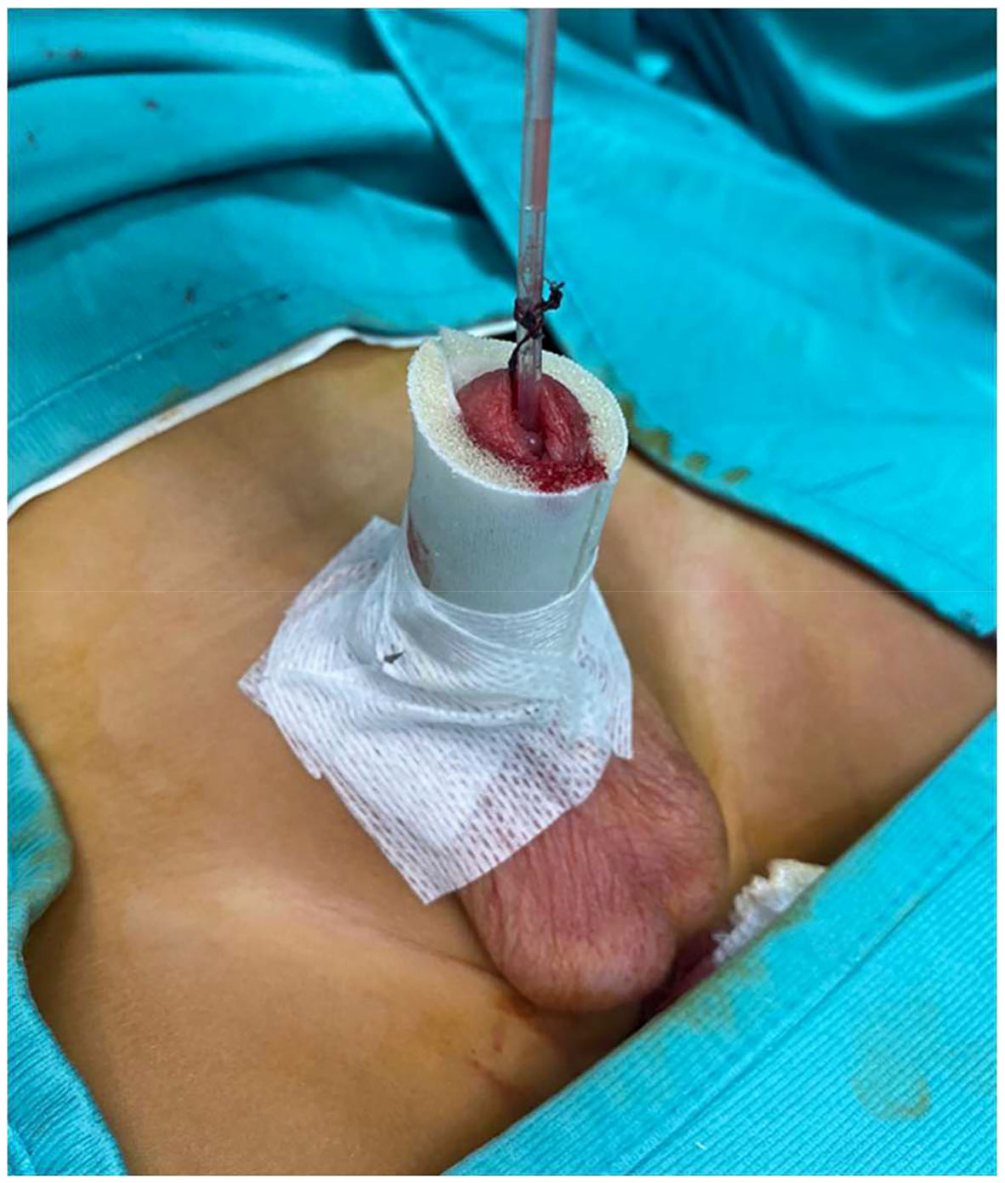
The final aspect of dressing using tubular finger device.

After completing the surgical correction of the hypospadias, the area was washed with saline and dried with gauze. In G2 patients, as already described in the previous study (15), a layer of oxygen-enriched oil-based gel was directly applied on a wet gauze composed by hyaluronic acid. This impregnated gauze was wrapped around the penis and subsequently covered by an elastic net bandage to obtain hemorrhage compressive effect. In G1 patients, a new medical device in the form of a tubular finger with a closed tip was adopted. The substrate of this device was polyurethane and polyester whereas, the inner part was coated with oxygen-enriched olive oil and lavender essential oil. The device was available in two different sizes, small (S) measuring 11 cm in length × 3 cm in diameter and large (L) measuring 11 cm × 4 cm. The S size was adopted in all the patients of our study. Before applying the device, it was cut to reduce its length in accordance with the penis size to ensure that the whole penis was covered. The closed tip of the device was cut off and the device was opened longitudinally to easily apply it and wrap the entire length of the penis (Figure 1). Finally, the device was stabilized onto the penis by closing the two edges using three interrupted stitches on the dorsal side of the penis taking care not to deform or crumple it excessively and ensuring that it sticked firmly to the entire wound area (Figure 2) and was fixed to the skin using adhesive tape (Figure 3).

The dressing was changed in all the patients of both groups on the 4th post-operative day (POD), then repositioned and finally removed on the 7th POD at the time of bladder catheter removal. After hospital discharge, all parents were asked to apply topically 2% eosin solution and oxygen-enriched oil-based gel without any bands or gauzes during the diaper change twice a day until completion of wound healing.

### Assessment of Outcome Measures

The follow-up evaluations were performed by one independent pediatric surgeon and one pediatric nurse, not involved in the operation and blinded to the patient group. The follow-up schedule included a clinical control at 7, 14, 21, 30, and 60 days postoperatively. At each control, the wound was observed and photographs of the penis were obtained to document the wound healing and the cosmetic result.

The primary endpoint of the study was to compare the two groups about the wound healing time defined as the time to return to normal structure and appearance of the penis following surgery. The wound healing was scored using the Southampton Wound Assessment Scale (SWAS) (15), that evaluated normal healing (grade 0), normal healing with mild bruising or erythema (grade 1), presence of erythema plus other signs of inflammation (grade 2), clear or hemo-serous discharge (grade 3), or major complications such as pus (grade 4) and deep or severe wound infection with or without tissue breakdown or hematoma requiring aspiration (grade 5) (Table 1).

**Table 1:** Southampton wound assessment scale (SWAS).

**Grade**	**Appearance**
0	Normal healing
1	Normal healing with:
a	Some bruising
b	Considerable bruising
c	Mild erythema
2	Erythema plus other signs of inflammation:
a	At one point
b	Around sutures
c	Along wound
d	Around wound
3	Clear or hemoserous discharge:
a	At one point only (<2 cm)
b	Along wound (>2 cm)
c	Large volume
d	Prolonged (>3 days)
**Major complication**	
4	Pus
a	At one point only (<2 cm)
b	Along wound (>2 cm)
5	Deep or severe wound infection with or without tissue breakdown; hematoma requiring aspiration

Furthermore, we evaluated the presence and duration of post-operative preputial edema. We measured the penile diameter before surgery and at fourth, seventh and fourteenth post-operative day (POD) to achieve an objective evaluation of swelling and its evolution at these timelines. The penile diameter was always measured 1-cm below the apex of reconstructed foreskin to obtain a homogeneous measurement in all patients.

Secondary outcome parameters included adverse reactions to the product, foreskin retractability, post-operative complications, level of nurses' difficulty to manage the dressing, and dressing costs in each group. Adverse skin reactions such as hypersensitivity or allergic reactions to the product were categorized by the researcher as absent, limited to the foreskin, or extended to other areas.

The retractability of the foreskin was also evaluated at > 30days postoperatively. At this time, the first retraction of the reconstructed prepuce was performed by the evaluating surgeon and continued by parents at home during daily hygienic care.

Post-operative complications including infections, foreskin dehiscence, meatal stenosis and urethrocutaneous fistula were also assessed and graded according to Clavien-Dindo classification (16).

The level of nurses' difficulty to manage the dressing was subjectively assessed by nurses on a 1–5 Likert-type scale, with 1 = very easy; 2 = easy; 3 = average; 4 = hard; 5 = very hard.

We performed a comparative analysis of all secondary outcome parameters between the two groups.

### Statistical Analysis

Statistical analysis was carried out using the Statistical Pack- age for Social Sciences (SPSS Inc., Chicago, Illinois, USA), version 13.0. Continuous variables were summarized and presented as median and interquartile range. The categorical variables were presented as absolute numbers and percentages.

The associations between qualitative variables were measured by the chi-square test and quantitative variables were measured with the parametric Student's *t*-test. *P* < 0.05 was considered statistically significant.

## Results

Sixty-four patients, with median age of 14 months (interquartile range, IQR 12–28), were included in the study. All the children wear the nappy at the time of surgery. The patients were randomized in two groups, each of 32 patients, according to the type of post-operative dressing: the treatment group (G1) included patients in whom the tubular finger oxygen-enriched oil inside-coated device, NOVOX TOUCH ® (MOSS SpA, Lesa, Novara, Italy), was adopted whereas, the control group (G2) included patients receiving the standard dressing consisting of elastic net bandage with application of oxygen-enriched oil-based gel NOVOX ® (MOSS SpA, Lesa, Novara, Italy).

There was no significant difference in the age at presentation (*p* = 0.44), the hypospadias degree (*p* = 0.33), the length of stay (*p* = 0.55) and the follow-up (*p* = 0.37) between the two groups.

The patients' demographics are summarized in Table 2.

**Table 2:** Comparative analysis of patients' demographics and outcomes between G1 and G2.

	**G1 Tubular oxygen-enriched oil inside-coated device *n = 32***	**G2 Elastic band + Oxygen-enriched oil-based gel *n = 32***	***P*-value**
Median patients age, years (IQR)	15 (13–28)	13 (12–26)	0.44
Balanic hypospadias, n (%)	12/32 (37.5%)	10/32 (31.2%)	0.33
Coronal hypospadias, n (%)	15/32 (46.9%)	17/32 (53.1%)	0.33
Subcoronal hypospadias, n (%)	5/32 (15.6%)	5/32 (15.6%)	0.33
Median length of stay, days (IQR)	8 (7–9)	8 (7–9)	0.55
Median follow-up, months (IQR)	11 (2–13)	10 (2–14)	0.37
Median length of surgery, minutes (IQR)	82 (70–105)	88 (75–110)	0.44
Median wound healing time, days (IQR)	14.2 (11–16)	18.5 (16–21)	0.001
Post-operative preputial edema, n (%)	3/32 (9.3%)	10/32 (31.2%)	0.001
Pre-operative mean ± SD penile diameter, cm	1.08 ± 0.17	1.07 ± 0.14	0.33
Post-operative mean ± SD penile diameter POD 4th, cm	1.14 ± 0.12	1.28 ± 0.15	0.001
Post-operative mean ± SD penile diameter POD 7th, cm	1.11 ± 0.11	1.22 ± 0.13	0.001
Post-operative mean ± SD penile diameter POD 14th, cm	1.07 ± 0.16	1.12 ± 0.11	0.03
Median duration of preputial edema, days (IQR)	6 (2–7)	10.5 (4–12)	0.001
Adverse skin reaction to the product, n (%)	0	0	n/a
Post-operative complications, n (%):	0	3/32 (9.3%)	0.001
- Foreskin dehiscence, n (%)	0	2/32 (6.2%)	0.001
- Urethroplasty and preputioplasty breakdown, n (%)	0	1/32 (3.1%)	0.001
Foreskin retractability at >30 days follow-up:			
- Retractile, n (%):	31/32 (96.9%)	31/32 (96.9%)	0.55
- Phimosis, n (%):	1/32 (3.1%)	1/32 (3.1%)	0.87
Nurses' scoring of dressing (l−5 Likert type scale: 1-very easy; 2-easy; 3-average; 4-hard; 5-very hard), n (IQR)	1.2 (1–3)	3.5 (2–4)	0.001
Median costs, eur (IQR)	55 (33–60)	87 (38–116)	0.001

*IQR, interquartile range; n/a, not applicable; SD, standard deviation; POD, post-operative day*.

The length of surgery ranged between 70 and 110 min (median 85). All the patients received analgesic therapy through an elastomeric infusion pump (tramadol 2 mg/kg) for the first 24 h post-operatively, followed by oral therapy (paracetamol 15 mg/kg/8h and tramadol 2 mg/kg/8h). The bladder catheter was removed on the 7th POD in all patients, who were discharged on the following day.

**Figure 4:**
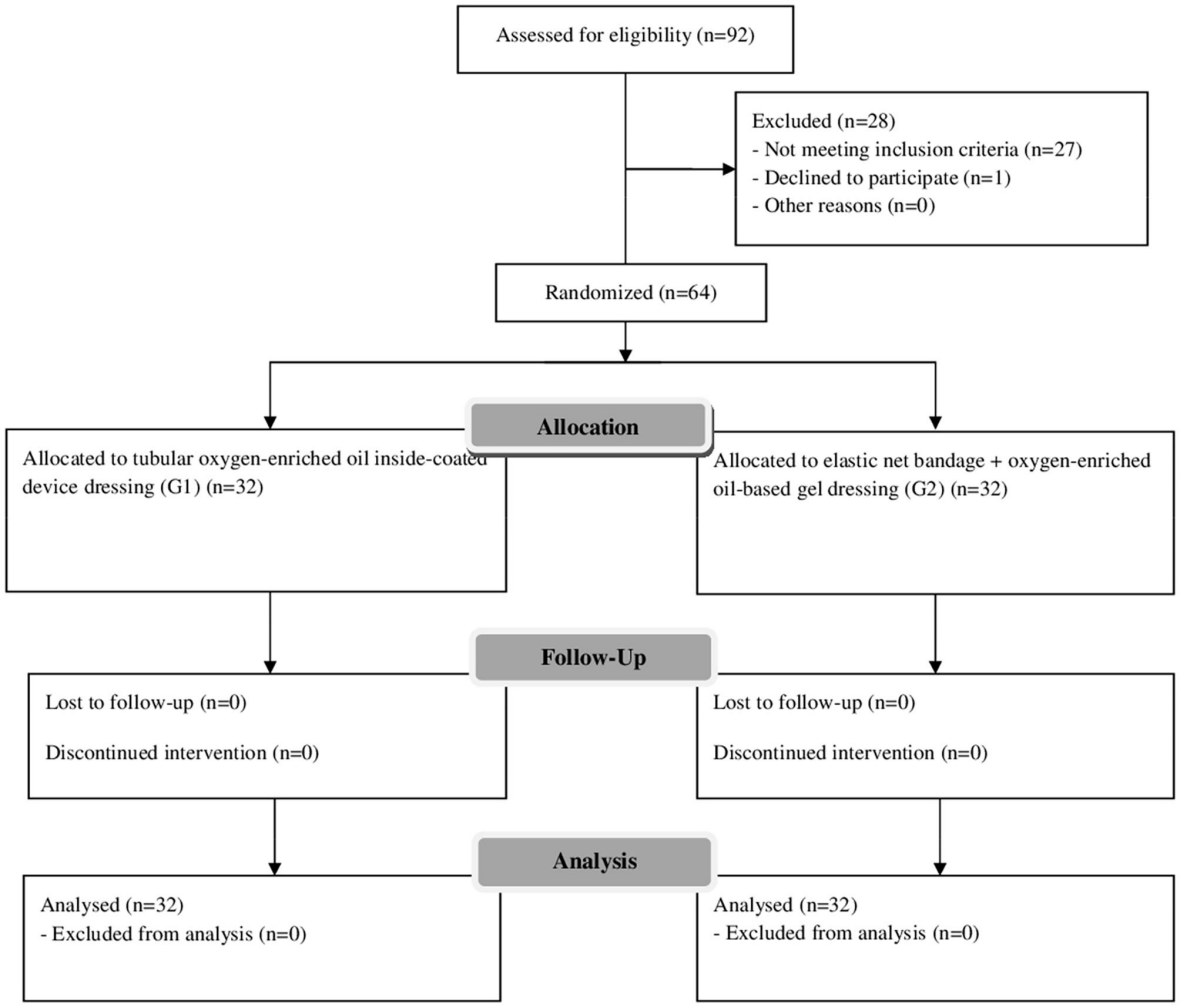
Patients' allocation.

**Figure 5:**
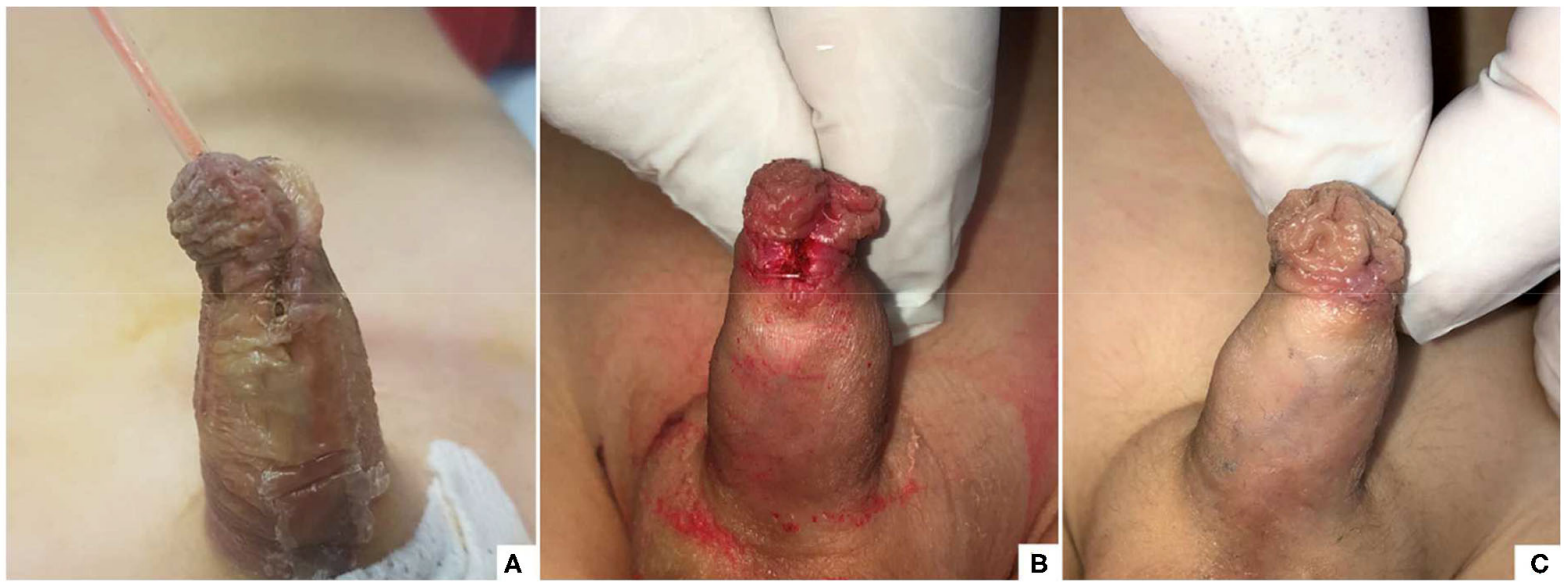
Wound healing in G1 at 7 days **(A)**, 14 days **(B)** and 30 **(C)** days post-operatively.

**Figure 6:**
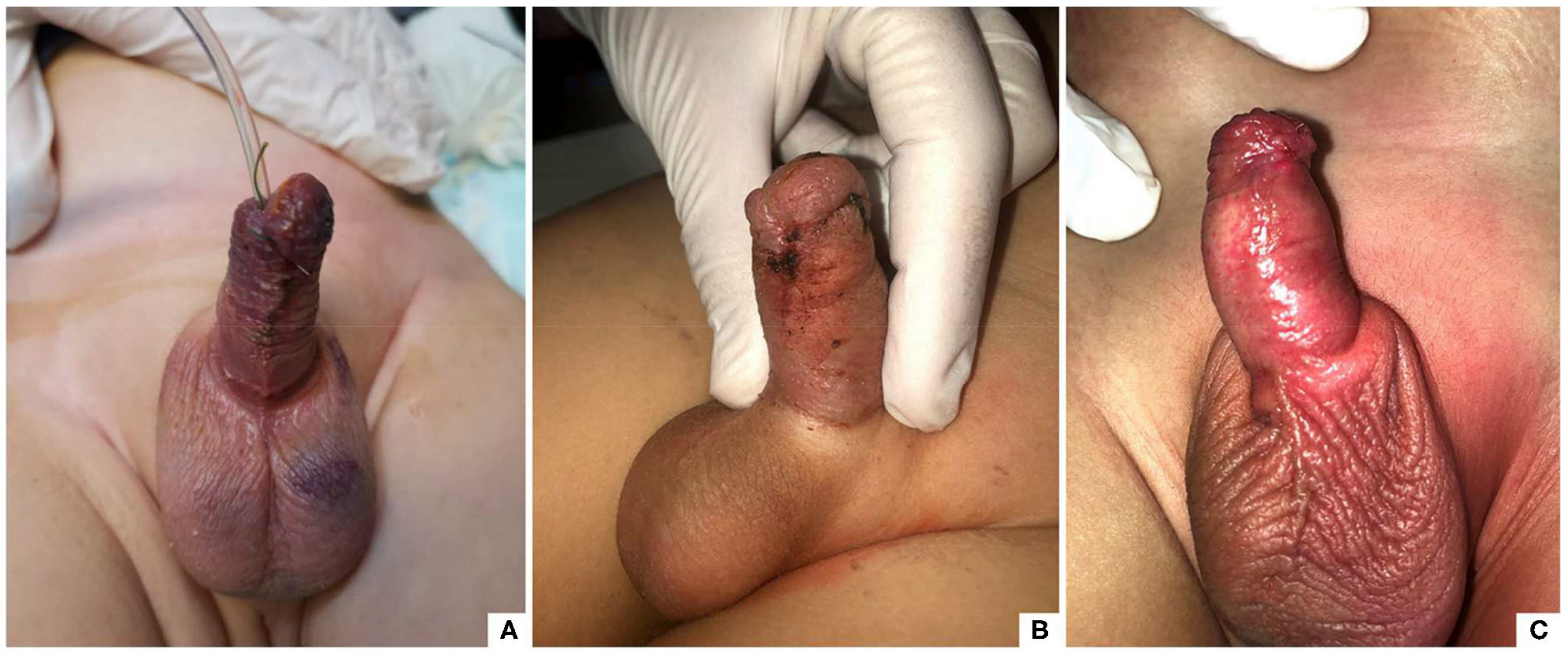
Wound healing in G2 at 7 days **(A)**, 14 days **(B)** and 30 **(C)** days post-operatively.

No patients were lost to follow-up (Figure 4). At clinical examination, the wound healing time was significantly shorter in G1 compared with G2 [14.2 vs. 18.5 days] (*p* = 0.001) (Figures 5, 6). Wound healing evaluation using SWAS reported a significantly higher rate of normal healing ( ≤ 1) in G1 compared with G2 at 7 days (84.4 vs. 59.4%), 14 days (100 vs. 68.8%), 21 days (100 vs. 84.4%) and 30 days (100 vs. 90.6%) follow-up (*p* = 0.001). No significant difference in SWAS scores was found between G1 and G2 (*p* = 0.33) at 60 days follow-up (Table 3).

**Table 3:** Southampton wound assessment scale (SWAS) scores in G1 and G2.

	**G1 Tubular oxygen-enriched oil inside-coated device** ***n=32***		**G2 Elastic band** **+** **Oxygen-enriched oil-based gel** ***n=32***		***P*-value**
	**≤1**	**>1**	**≤1**	**>1**	
7 days follow-up	84.4% (27/32)	15.6% (5/32)	59.4% (19/32)	40.6% (13/32)	0.001
14 days follow-up	100% (32/32)	0	68.8% (22/32)	31.2% (10/32)	0.001
21 days follow-up	100% (32/32)	0	84.4% (27/32)	15.6% (5/32)	0.001
30 days follow-up	100% (32/32)	0	90.6% (29/32)	9.4% (3/32)	0.001
60 days follow-up	100% (32/32)	0	100% (32/32)	0	0.33

Post-operative preputial edema incidence was significantly lower in G1 (3/32, 9.3%) compared with G2 (10/32, 31.2%). Post-operative mean ± standard deviation (SD) penile diameter (cm) was significantly lower in the treatment group (G1) compared with the control group (G2) at 4th POD (1.14 ± 0.12 vs. 1.28 ± 0.15) (*p* = 0.001), 7th POD (1.11 ± 0.11 vs. 1.22 ± 0.13) (*p* = 0.001) and 14th POD (1.07 ± 0.16 vs. 1.12 ± 0.11) (*p* = 0.03). The median duration of preputial edema was significantly shorter in G1 compared with G2 (6 vs. 10.5 days) (*p* = 0.001).

No adverse skin reactions or allergies to the product occurred in both groups. The evaluation of foreskin retractability showed no significant difference about incidence of post-operative phimosis between the two groups (*p* = 0.87). The post-operative complications rate was significantly lower in G1 (0%) compared with G2 [(3/32 Clavien IIIb) 9.3%] (*p* = 0.001). In two G2 patients (6.2%), foreskin dehiscence occurred whereas, in one G2 patient (3.1%) breakdown of urethroplasty and preputioplasty occurred due to scratching injuries caused by the child on the 5th POD.

The dressing management was subjectively evaluated by nurses to be easier in G1 patients (median score 1.2) compared with G2 ones (median score 3.5) (*p* = 0.001).

The median treatment costs were significantly lower in G1 (55 eur) compared with G2 (87 eur) (*p* = 0.001).

The comparative analysis of outcomes between G1 and G2 is summarized in Table 2.

## Discussion

Hypospadias repair is one of the most common surgical procedures performed by pediatric surgeons (17). Limited evidence is available regarding several aspects of surgical management of hypospadias, including details of surgical technique, type of suture, indications for foreskin reconstruction, type and length of urinary diversion, and post-operative dressing (18–20).

One of the most controversial aspects of hypospadias surgery is the choice of an appropriate wound dressing (1). Multiple dressings have been previously described (3–10); however, there is no evidence in the current literature about the best method for post-operative dressing following hypospadias repair (2). The choice of an adequate dressing is crucial because the success of the surgical procedure may be influenced by post-operative management of the penis. The dressing is particularly important in those patients undergoing preputioplasty and urethroplasty, who may develop a considering preputial edema in the post-operative period. This may cause a high tension on the suture line and increase the risk of foreskin and urethra dehiscence.

The ideal hypospadias dressing should be rigid but at the same time flexible, elastic, resistant, it should keep the penis straight and provide an effective pressure on the wound (11). In addition, hypospadias dressing should present minimal adverse reactions when in contact with tissues, protect the penis against the contamination by pathogens and also against the traumatic injuries, frequently caused by the child's scratching on the wound, that may occur in the early post-operative period. Finally, the ideal dressing should be easy and painless to change and to remove (12–14).

We discovered incidentally this new device, that was originally created to treat fingers or toes wounds, ulcers, injuries and burns, and its shape and size caught our attention. In fact, it was cylindric, with a closed tip and was long 11 cm and large 3 cm in the size S. This new device seemed to fit all the characteristics of an ideal hypospadias dressing, because it was stable and kept the penis straight with a lower incidence and duration of post-operative swelling compared with the standard dressing, as reported in our study. It was an effective mechanical barrier through a double mechanism: first, the non-impregnated outside of the device was fairly water-repellent and protected the penis against urinary and fecal contamination but also against traumatic injuries, caused by the child's scratching on the wound area, as happened in our experience. Second, the inner part of the device, coated with oxygen-enriched oily gel, created a micro-environment, that was unfavorable to the proliferation of the pathogens commonly found on skin lesions, allowing activation of the microcirculation and thanks also to the protective, barrier and soothing action of oxygen-enriched olive oil (21–26). In fact, seepage of small amounts of oxygen-enriched oil from the edges of the dressing allowed the oil to be in direct contact with the wound and the pungent smell was an intrinsic characteristic, indicating the release of reactive oxygen species.

The oxygen-enriched oil-based gel we adopted in the study comes in the form of an oily gel, derived from extra virgin olive ozonated oil, hyperoxidized and standardized in peroxides. Regarding the mechanism of action, the application of the gel promotes the physiological repair of the wound, having a film-forming protective action and improves re-epithelialization, promoting the proliferation of fibroblasts. The beneficial effects of ozone on wound healing are related to the reduction of microbial infection, debridement effect, modulation of the inflammatory phase, stimulation of angiogenesis as well as biological and enzymatic reactions that favor oxygen metabolism improving wound healing (23). It acts as insulator, producing heat that results in local peripheral vasodilation, increased blood flow, oxygenation and cellular metabolism, accelerating the healing process. The effect on the skin is due to its reaction with the polyunsaturated fatty acids and traces of water present in the upper layer of the dermis, generating reactive oxygen species (ROS) and lipo-oligopeptides, among which is H_2_O_2_. ROS are the most effective natural agents against antibiotic-resistant pathogens and favor the degradation of organic material which could disturb the healing process. In addition, it improves metabolism and immune functions (23). Ozone therapy activates the production of some nuclear factors to induce cytokines transcription, such as IL-2, TNFα, IL-6, IFNγ and IL-8, participating in the immune response of our body. Furthermore, ozone enhances a higher expression of growth factors TGF-β and vascular endothelial growth factor (VEGF), which play important roles in the wound repair process. In this way, remodeling of the extracellular matrix (ECM) begins and collagen fibers proliferate and reorganize into a stronger network (27). In these situations, ozone promotes the release of nitric oxide (NO), endothelium-independent vasodilator, which increases blood circulation for tissue remodeling (27).

For all these reasons, this new device was very effective in our series in promoting a faster healing of penis wound compared with the standard dressing method. The beneficial effects on the wound healing were also associated with a better post-operative outcome in our study. In fact, post-operative complications rate was statistically significant lower in G1 patients compared with G2 ones (*p* = 0.001). Furthermore, penile diameter measurements at different timelines proved that entity and duration of post-operative swelling were objectively decreased using the new dressing method.

The clinical use of the device was easier compared with the standard dressing method for different reasons, as subjectively assessed by nurses. First, the tubular device was fairly water-repellent and protected the penis against urinary and fecal contamination whereas, the elastic bandage was not water-repellent and was more frequently exposed to contamination with urines or stools, especially of fluid-consistency, and needed more frequently to be changed. Second, the dressing change was also easier using the tubular device compared with the standard method; in fact, it only needed to remove the posterior anchoring stitches and the device was detached off the penis. A new tubular device was then re-applied and closed on the dorsal side of the penis using 2–3 pieces of adhesive tape without any discomfort for the child. The device never sticked to the penis thanks to the constant release of oil from the inner coated side. Conversely, the elastic bandage frequently sticked to the penis because the layer of oxygen-enriched oil-based gel directly applied on the wound was quickly absorbed by tissues.

The use of the device was clinically safe because it was a preparation for topical use and had no systemic effects. Additionally, the topical use in our series did not report any hypersensitivity or allergic or other serious skin reactions to the dressing.

Finally, the cost analysis showed that the new tubular device for hypospadias dressing was also cost-effective. The lower costs of wound treatment in G1 patients were related to the faster healing process in such patients, who required a shorter treatment following hospital discharge, compared with the G2 patients.

One the main limitations of the study is the short follow-up time which could influence the overall surgical complications rate as previous papers (28, 29) have shown urethroplasty complications to develop well-passed the 60 days follow-up of our paper. We must also consider that in other countries such as the United States (US) most distal hypospadias are done as outpatient. As reported by Snodgrass et al. (30), a simple clear plastic wrap (Tegaderm) was used as dressing for a few days postoperatively and their complications rate tended to be around 8%. The lack of a control group using a similar type of dressing in our study could be considered a further limitation.

Additionally, the way this dressing is being described would possibly suggest a limit for its utilization in the outpatient setting. We preferred that the patients remained in the hospital after the operation with the aim to assess different aspects of this dressing during the hospital stay such as the tolerability by patients and the ease of management by nurses and parents. However, this does not mean that it cannot be adopted in the outpatient setting. Probably, it is better than the previous dressing methods because it does not require any manipulation by parents. It could be conceived that patients are operated as outpatient and discharged following the operation and then come back to the hospital for dressing change and thereafter for catheter removal.

Another limitation is represented by the restrictive inclusion criteria, limited to distal hypospadias repairs. The next step will be to test this new dressing method in a larger number of patients with a longer follow-up and include also proximal hypospadias to validate these preliminary results.

In conclusion, based upon the preliminary results of this study, post-operative dressing using tubular finger oxygen-enriched oil inside-coated device was highly effective, easy to manage, cheaper and associated with a lower rate of foreskin and urethral complications compared with the standard dressing method in pediatric patients undergoing distal hypospadias repair. It was also cost-effective and clinically safe without allergy or intolerance to the product.

## Data Availability Statement

The original contributions presented in the study are included in the article/supplementary material, further inquiries can be directed to the corresponding author.

## Author Contributions

CE contributed conception and design of the study and wrote the first draft of the manuscript. VC, FD, MCe, GE, FC, MCa, AC, and ME organized the database and wrote sections of the manuscript. All authors contributed to manuscript revision, read and approved the submitted version.

## Conflict of Interest

The authors declare that the research was conducted in the absence of any commercial or financial relationships that could be construed as a potential conflict of interest.
